# The Hand the Servant of the Brain

**Published:** 1900-06

**Authors:** E. A. Bogue

**Affiliations:** New York


### THE HAND THE SERVANT OF THE BRAIN.[1]

BY DR. E. A. BOGUE, NEW YORK.

Mr. President and Gentlemen,—By making the hand serve the brain, manufacturing nations have uniformly come to the front.

In the fifteenth and sixteenth centuries Spain was the most powerful nation in Europe, and at that time the strongest in her manufactures, but she came to rely upon the force of arms for her supremacy, and adopted the Roman idea that it was demeaning to work with the hands. She discouraged handiwork and drove the Moors, her Mohammedan workers, out of her borders, while her better classes entered the army and the church. Her position to-day is known of all men; it is not magnanimous to dwell upon it. England and the Anglo-Saxon races have encouraged manufactures until the disseminated products of their handiwork may be found in the uttermost corners of the habitable globe. When, finally, the American idea was found at Versailles by the German conquerors after the victory at Sedan, Germany speedily set about organizing another victory, and this time it was an industrial one, the proof of which we see every time we read the legend, "Made in Germany."

Turning now to our own calling, we remark that in France the first steps were taken to make of us a distinct profession between the years 1306 and 1311, for it was then that the separation took place between the doctors and the barbers practising surgery, including tooth-pulling.

The beginning of so-called American dentistry was in 1775 or 1776, when Mr. Robert Woffendale, educated as a dentist by Thomas

---

[1] Read before the union meeting of The New York Institute of Stomatology and the American Academy of Dental Science, Boston, March 21, 1900.

Bardmore, dentist to George III., arrived in America, so that the first American dentist was an Englishman.

Among the first who followed him was Joseph Lemaire, a French dentist; then Dr. James Gardette, another Frenchman, and then Isaac Greenwood, another Englishman, who took up his residence in Boston and made teeth for the first President of the United States, and though he did not receive the public appointment of dentist to the President, he yet became and has remained celebrated because of it.

The first dental college was chartered at Baltimore in 1839, and held its first session in 1840. From that time to this the history of American dentistry has been a history of the adaptation of means to ends, a history of constant inventions and their application in actual practice. The most distinguished men practising dentistry as a specialty have not been, as a rule, men who were graduated in medicine and failing in that took up dentistry, but men whose fingers had been taught an art the limits of which they had perceived as they undertook to practise it, and those limits had proved an incentive to further and continued efforts.

For many years the American mode of practice was either more conservative or more successful than others, for the American dentist did not hesitate to work with his hands. As a result, the American dentist abroad was sought after by those classes in society having both intelligence and money. The American dentist has not been tramelled either by the traditions of society, for he had not any, or of the medical school, for he did not graduate there, nor yet by an *esprit-de-corps,* for the "corps" did not exist, but as he had been brought up by hand to encounter difficulties and to conquer them with his hands and his head, he went right on in that same way in his chosen profession.

When he found that his calling was dignified because it was exercised upon the human body, he straightway betook him to the medical school that he might get additional knowledge.

The question, therefore, is upon us to-day, What is the best method of training a dental surgeon for his peculiar work?

Our professional colleagues in France and England have adopted different ways. France began by establishing technical schools, and had reached a point of respectable success in her teachings when a class of men who called themselves physicians of the mouth ("médecins de la bouche") and were graduates in medicine succeeded in having such laws passed that no one could thenceforward practise dentistry in France without being submitted to an examination. The examining boards established by this law are composed of medical men. Almost immediately practical work in these French schools fell off. Instead of seeking to acquire sufficient technical skill to enable them to perform ordinary dental operations, the students set to work to acquire enough theoretical knowledge to answer the questions propounded by the medical men on the boards of examination. The clinics dwindled, and the skill of the candidates also. This comes from putting theory first and practice afterwards.

Our English friends began by admitting into the College of Surgeons dental students who were able to pass the preliminary examinations. These students went through portions of the same course as that taken by the general surgeon, with divergence enough to allow them to acquire such knowledge in the dental hospital as would enable them to pass as licentiates of dental surgery, or, if they wished to rank well, and could spend the necessary time and money, they graduated as surgeons and gained the L.D.S. besides. Many of the better ones from among these students came to America to acquire the manipulative skill that they could not procure at home, until finally England passed her dental law, which resulted in keeping most of them in the home schools. It has seemed to me that the criticism of Herbert Spencer upon certain examination papers once sent to him would be very applicable to the examinations, not only of the English and French examining boards, but at the present time very largely to our own as well,—viz., "They are drawn up with the exclusive view of testing *acquisition* rather than *power*. . . . The more important thing to be ascertained is not the quantity of knowledge which a man has taken in, but the ability he shows to use the knowledge he has acquired."

In the February number of the INTERNATIONAL DENTAL JOURNAL an editorial article advocates the addition of a fourth year to the course of instruction for a dentist, and adds: "It is either four years or a reduction in the curriculum. It must be evident to all experienced teachers that we are graduating men of partial culture in many things and experts in nothing. The practical branches are suffering as never before, and this in spite of the time devoted to technical work."

Two distinguished members of the dental hospital of the London Medical School were to have been with us this evening. They inform me that after great efforts a few of the more earnest ones among them have succeeded in establishing, in addition to the theoretical instruction heretofore given, a more extended technical training in the dental hospital to which they are attached than has heretofore been attainable in England except by private tuition, so that now the English student may take his technical training with his theoretical, as the French student formerly could. What, then, considering the steps along which we have come, is the surest way to obtain a profound theoretical and practical knowledge of our calling? We are taught by the successes of American dentistry as well as by the acknowledged shortcomings in the teachings of the other nations.

It is not generally recognized that man has several ways of expressing himself, and that sculpture, architecture, and finger craft, directed by a qualified and instructed brain, are more durable forms of expression and often more productive of good results than oral speech.

He who can do a useful thing well takes delight in the doing, and he who has joy in the doing is sure to be doing well and is going on towards perfection.

How many students are gradated from our literary and professional colleges who have no means of pronouncing themselves? How many have their heads full of knowledge, but are unable to turn their hands to a single useful thing?

The cowboy graduates of Oxford and Cambridge, whom I have seen, and of Yale and Harvard, of whom I have heard, give evidence of the hiatus in our educational methods.

The feeling of strong mental powers, but of expressional helplessness, which oppresses young men just out of college would vanish if manual training had taught them how to express themselves with their hands. Manual training properly defined would result in the development of the understanding and expressing man.

I advocate, therefore, the continuation of manual training from the kindergarten to the doctor's degree, confident that, in whatever calling a man may engage, success consists in being useful to somebody else than himself. The purely selfish man will not succeed.

He who receives a suffering patient with the mental question, "How much is there in it for me?" will never acquire manual dexterity, though he may succeed in writing a page as illegibly as

Choate himself. He will never become a great surgeon. He will never rank among the best oral surgeons.

We should commence our professional education by training the hand. We should teach the art, and with it the science which underlies that art, which enlightens the mind that practises it and guides the hand that accomplishes it, and as the mind opens more and more to comprehend the work that is being done, it will seek ways out of the difficulties that beset us in our daily work by endeavoring to understand more thoroughly the physical laws governing the tissues with which we have to deal.

Professor Egleston was unquestionably right when he insisted in the foundation of the Columbia University School of Mines that the best channel by which the mind could be enlarged and enlightened and become able to direct its servant,—the hand,—was through manual training and the complementary studies that necessarily accompany it.

In insisting upon such professional training for practitioners of our specialty, I do not wish to forget the remarks of Professor Blackie, of the University of Edinburgh, who says, "The merely professional man is always a narrow man. . . . In society the most accomplished man of mere professional skill is often a nullity; he has sunk his humanity in his dexterity. He is a leather dealer, and can talk only about leather; a student, and smells fustily of books, as an inveterate smoker does of tobacco. . . . The most exact professional drill will omit to teach him the most interesting and the most important part of his own business,—that part, namely, where the specialty of the profession comes directly into contact with the generality of human notions and human sympathies. The best preservatives against the cramping force of merely professional study are the healthy influences of society, travel, and a familiarity with great writers,—especially poets and historians,—whose thoughts 'make rich the blood of the world' and enlarge the platform of sympathetic intelligence."

We have now upon our side the recent action of the German Emperor, who has given to the three Prussian High Schools of Technology of Berlin, Aix-la-Chapelle, and Dortmund the right of conferring the degree of "Doctor Ingenieur," so that the young engineer, chemist, or architect may now have the title which in Germany is of so much value both in a professional and social sense.

This gives an added dignity to technical training, and is an acknowledgment from a pretty high authority of the value of those men who can do something with their hands.

